# BCG and BCG/DNAhsp65 Vaccinations Promote Protective Effects without Deleterious Consequences for Experimental Autoimmune Encephalomyelitis

**DOI:** 10.1155/2013/721383

**Published:** 2013-10-29

**Authors:** Sofia Fernanda Gonçalves Zorzella-Pezavento, Clara Pires Fujiara Guerino, Fernanda Chiuso-Minicucci, Thais Graziela Donegá França, Larissa Lumi Watanabe Ishikawa, Ana Paula Masson, Célio Lopes Silva, Alexandrina Sartori

**Affiliations:** ^1^Departamento de Microbiologia e Imunologia, Instituto de Biociências, Universidade Estadual Paulista (UNESP), 18618-970 Botucatu, SP, Brazil; ^2^Departamento de Bioquímica e Imunologia, Universidade de São Paulo (USP), 14049-900 Ribeirão Preto, SP, Brazil

## Abstract

A prime-boost strategy conserving BCG is considered the most promising vaccine to control tuberculosis. A boost with a DNA vaccine containing the mycobacterial gene of a heat shock protein (pVAXhsp65) after BCG priming protected mice against experimental tuberculosis. However, anti-hsp65 immunity could worsen an autoimmune disease due to molecular mimicry. In this investigation, we evaluated the effect of a previous BCG or BCG/pVAXhsp65 immunization on experimental autoimmune encephalomyelitis (EAE) development. Female Lewis rats were immunized with BCG or BCG followed by pVAXhsp65 boosters. The animals underwent EAE induction and were daily evaluated for weight loss and clinical score. They were euthanized during recovery phase to assess immune response and inflammatory infiltration at the central nervous system. Previous immunization did not aggravate or accelerate clinical score or weight loss. In addition, this procedure clearly decreased inflammation in the brain. BCG immunization modulated the host immune response by triggering a significant reduction in IL-10 and IFN-**γ** levels induced by myelin basic protein. These data indicated that vaccination protocols with BCG or BCG followed by boosters with pVAXhsp65 did not trigger a deleterious effect on EAE evolution.

## 1. Introduction

Tuberculosis (TB) is an infection caused by *Mycobacterium tuberculosis* and this disease remains one of the most important causes of death worldwide [1, 2]. Factors as coinfection with human immunodeficiency virus and emergence of drug resistance in *M. tuberculosis* strains have hampered TB control [3, 4].

The only available vaccine against TB is the attenuated *M. bovis* Bacillus Calmette-Guérin (BCG) that is recommended by the World Health Organization for all infants under 1 year of age. Around 100 million newborn children receive this vaccine and the global vaccine coverage is estimated to be 80% [5, 6]. In spite of this extensive use, numerous well-documented trials showed significant variation, from 0 to 80%, in BCG protective efficacy [7]. This has been attributed to variability in BCG vaccine strains and environmental factors as well as host genetic background [8, 9].

Although BCG seems to provide protection against disseminated tuberculosis in newborns and children, the induced immunity wanes with age, resulting in insufficient protection against adult pulmonary TB [10, 11]. In this context, there is a great interest in the development of new vaccines against TB. Numerous alternative living and nonliving putative TB vaccines are being lately tested [12–14]. Experimental evidence indicated that DNA vaccines, due to their ability to induce a strong Th1 type of response, could contribute to TB control. DNA constructs encoding mycobacterial antigens as 65 kDa heat shock protein (hsp65), Ag85A, Ag85B, and PstS3 induced significant protective immunity [15–17]. We previously demonstrated that a DNA plasmid encoding the *Mycobacterium leprae* 65 kDa heat shock protein exhibited prophylactic [18] and therapeutic activity in a TB murine model [19, 20]. In spite of these successful results with homologous vaccination protocols, heterologous prime-boost regimens, capable to increase BCG or rBCG efficiency, are considered more promising for future TB control [11]. In this context, we observed that pVAXhsp65 and BCG similarly primed neonate mice for a strong immune response to pVAXhsp65 boosters administered later, at the adult stage [21]. Prime-boost strategies combining these two vaccines were also able to protect mice and guinea pigs against experimental TB [22, 23].

 One of the arguments against the potential use of pVAXhsp65 alone or combined with BCG is the fact that hsp65 from *M. leprae*, whose gene is inserted in this DNA vaccine, presents a high degree of homology with its equivalent mammalian protein [24]. Theoretically, an anti-hsp65 immunity started with BCG and boosted by pVAXhsp65 could provoke or worsen an autoimmune disease. In support of this argument, many studies revealed immune response against bacterial hsp65 in diabetes [25], atherosclerosis [26], arthritis [27], and multiple sclerosis [28]. In addition, CpG motifs that are frequently present in bacterial plasmid vectors could trigger or exacerbate an autoimmune response [29, 30]. Even though BCG has been described as a safe vaccine, a few publications suggested its implication as a possible trigger of autoimmunity [31, 32]. In this context, the present study was designed to investigate if a vaccination protocol against TB, using BCG alone or a priming with BCG followed by boosters with pVAXhsp65, could aggravate or accelerate experimental autoimmune encephalomyelitis.

## 2. Material and Methods

### 2.1. Experimental Design

Female Lewis rats were immunized with BCG or with BCG plus pVAXhsp65. The animals underwent EAE induction by immunization with myelin basic protein (MBP). The effect of BCG or BCG/pVAXhsp65 on EAE was evaluated by clinical follow-up (weight variation and clinical score), histopathological analysis of the brain and lumbar spinal cord, and also by cytokine production. Nonimmunized and pVAX (empty vector) injected animals were included as control groups.

### 2.2. Animals

Female Lewis rats (4–6 weeks old) were purchased from CEMIB (UNICAMP, São Paulo, SP, Brazil). The animals were fed with sterilized food and water *ad libitum *and were manipulated in accordance with the ethical guidelines adopted by the Brazilian College of Animal Experimentation. All experimental protocols were approved by the local Ethics Committee (Ethics Committee for Animal Experimentation, Medical School, Universidade Estadual Paulista).

### 2.3. Genetic Vaccine Construction and Purification

The vaccine pVAXhsp65 was derived from the pVAX vector (Invitrogen, Carlsbad, CA, USA), previously digested with BamHI and NotI (Gibco BRL, Gaithersburg, MD, USA) by inserting a 3.3 kb fragment corresponding to the *M. leprae* hsp65 gene and the CMV intron A. The empty pVAX vector was used as a control. DH5*α* *E. coli* cells transformed with plasmid pVAX or the plasmid carrying the hsp65 gene (pVAXhsp65) were cultured in LB liquid medium (Gibco BRL, Gaithersburg, MD, USA) containing kanamycin (100 *μ*g/mL). The plasmids were purified using the Concert High Purity Maxiprep System (Gibco BRL, Gaithersburg, MD, USA). Plasmid concentrations were determined by spectrophotometry at *λ* = 260 and 280 nm by using the Gene Quant II apparatus (Pharmacia Biotech, Buckinghamshire, UK).

### 2.4. Immunization with BCG and pVAXhsp65

Lewis rats were immunized with BCG or with BCG followed by pVAXhsp65 boosters. The *M. bovis* BCG Moreau-Rio de Janeiro (2 to 10 × 10^5^ UFC) was inoculated a single time by subcutaneous route at the base of the tail. pVAXhsp65 was injected twice (300 *μ*g each) by intramuscular route (quadriceps muscle), being the first dose administered 15 days after BCG and the second one 15 days later. Control groups received the same volume of saline or the same concentration of pVAX (empty vector).

### 2.5. EAE Induction and Evaluation

EAE was induced as previously described [33]. Briefly, 15 days after the last DNA immunization, EAE was induced by inoculation of 25 *μ*g of MBP (Sigma Aldrich, St. Louis, MO, USA) emulsified with complete Freund's adjuvant (CFA) containing 5 mg/mL of *Mycobacterium butyricum*, in the hind left footpad. Animals were daily evaluated for weight loss and clinical score. Signs of disease were graded as 0 (zero): no disease; 1: loss of tonicity in the distal portion of the tail; 2: total loss of tail tonicity; 3: hind limb weakness (partial paralysis); 4: complete hind limb paralysis and urinary incontinence; and 5: moribund.

### 2.6. Evaluation of Inflammatory Infiltrates in the Central Nervous System (CNS)

A histological analysis was performed in the CNS at the 20th day after EAE induction, that is, during the recovery phase. After euthanasia and blood withdrawal, brain and lumbar spinal cord samples were removed and fixed in 10% formaldehyde. Tissues were dehydrated in graded ethanol and embedded in a 100% paraffin block. Five-micron thick sections were mounted over glass slides and stained with hematoxylin and eosin. The perivascular inflammatory infiltrates present in the brain and lumbar spinal cord were quantified by using the KS300 software (Carl-Zeiss, Germany). The images were analyzed with a computer-assisted image system based on a Nikon Microphot-FXA optical microscope connected, via a Sony Exwave HAD video camera, to a computer. Total section area of each sample was measured to avoid any interanimal variance. Further, perivascular mononuclear infiltrated areas of the whole sections were assessed by point-counting morphometry, as described elsewhere [34]. The values were expressed as *μ*m^2^ of cellular infiltrate per mm^2^ of organ section (*μ*m^2^/mm^2^).

### 2.7. Cell Culture Conditions and IFN-*γ* and IL-10 Production

Control and immunized rats (BCG or BCG/DNA) were euthanized eight weeks after initial BCG immunization. Lymph node (popliteal + inguinal) and spleen cells were collected and adjusted to 2.5 × 10^6^/mL and 5 × 10^6^/mL, respectively. Cells were cultured in complete RPMI medium (RPMI supplemented with 5% FCS, 20 mM glutamine, and 40 IU/mL of gentamicin), in the presence of 10 *μ*g/mL of rhsp65, 10 *μ*g/mL of MBP, or 5 *μ*g/mL of Concanavalin A (Sigma Aldrich). IFN-*γ* levels were assayed in lymph node cell cultures whereas IL-10 production was evaluated in spleen cell cultures. Cytokine levels in culture supernatants were evaluated 48 h later by ELISA according to manufacturer's instructions (R&D Systems). Briefly, ninety-six well plates (NUNC) were coated with capture antibodies for IFN-*γ* (DY 585) or IL-10 (DY 522) diluted in PBS at 2 *μ*g/mL and 4 *μ*g/mL, respectively. Plates were incubated overnight and then blocked during 2 h with 1% albumin in PBS. Standard rat cytokines and culture supernatants were added and the plates were incubated during 2 h. Biotinylated anti-IFN-*γ* and anti-IL-10 were added (150 and 100 ng/mL, resp.) and plates were incubated for additional 2 h at room temperature. After incubation at room temperature for 30 minutes with streptavidin, the plates were revealed by adding H_2_O_2_ + OPD (Sigma Aldrich, St. Louis, MO, USA). Color development was stopped with H_2_SO_4_ and optical density was measured at 492 nm.

### 2.8. Statistical Analysis

Statistical analysis was performed using SigmaStat statistical software (Jandel Corporation, San Rafael, CA, USA). Cytokine data was expressed as mean ± standard error of the mean (SEM) and tested for statistical significance by Kruskal-Wallis nonparametric test or one-way ANOVA followed by Tukey's test. Morphometric analysis of the brain and lumbar spinal cord was tested by one-way ANOVA followed by Holm-Sidak method. A *P* value of less than 0.05 was considered statistically significant.

## 3. Results

### 3.1. EAE Evolution is Not Aggravated by Previous Immunization with BCG or BCG/pVAXhsp65

**Figure 1:**
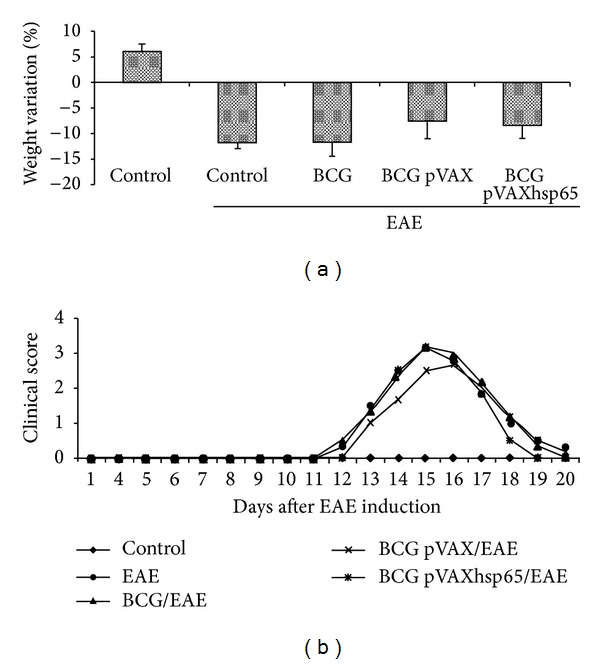
Effect of previous immunization with BCG and BCG/pVAXhsp65 in clinical EAE development. Female Lewis rats were immunized with BCG alone or with BCG followed by pVAXhsp65 boosters and then underwent EAE induction by inoculation of MBP emulsified with CFA. Animals were daily evaluated for weight variation (a) and clinical score (b). Data are presented by mean ± SEM for 4–6 rats.

EAE development caused, as expected, a weight loss that varied from 12 to 15% of the original weight. Previous vaccination with BCG alone, associated with pVAXhsp65 or with the empty vector, did not affect weight loss (Figure 1(a)). In the control EAE group, that is, in the sick group that was not previously immunized against TB, clinical symptoms appeared 11 or 12 days after MBP inoculation and clinical scores reached 2.5 to 3.0 (Figure 1(b)). A very similar clinical evolution was observed in the groups that were previously immunized with BCG, BCG/pVAXhsp65, or BCG/pVAX.

### 3.2. Both Vaccination Strategies Decreased Inflammation in the Brain

**Figure 2:**
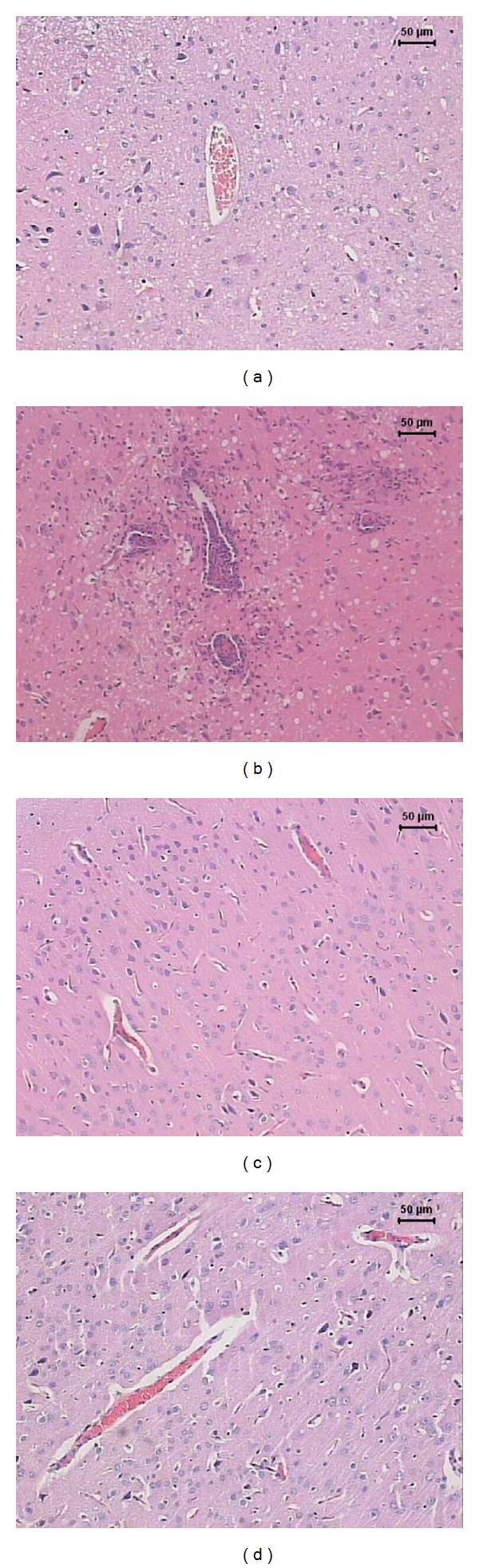
Effect of tuberculosis vaccines in brain inflammation associated with EAE. Female Lewis rats were immunized with BCG alone or with BCG followed by pVAXhsp65 boosters and then underwent EAE induction by inoculation of MBP emulsified with CFA. Animals were euthanized during the recovery phase (20th day after MBP injection) and the brains were removed and further stained by haematoxylin and eosin. Normal control (a), EAE control (b), rats immunized with BCG before EAE (c), and rats immunized with BCG/pVAXhsp65 before EAE induction (d).

**Figure 3:**
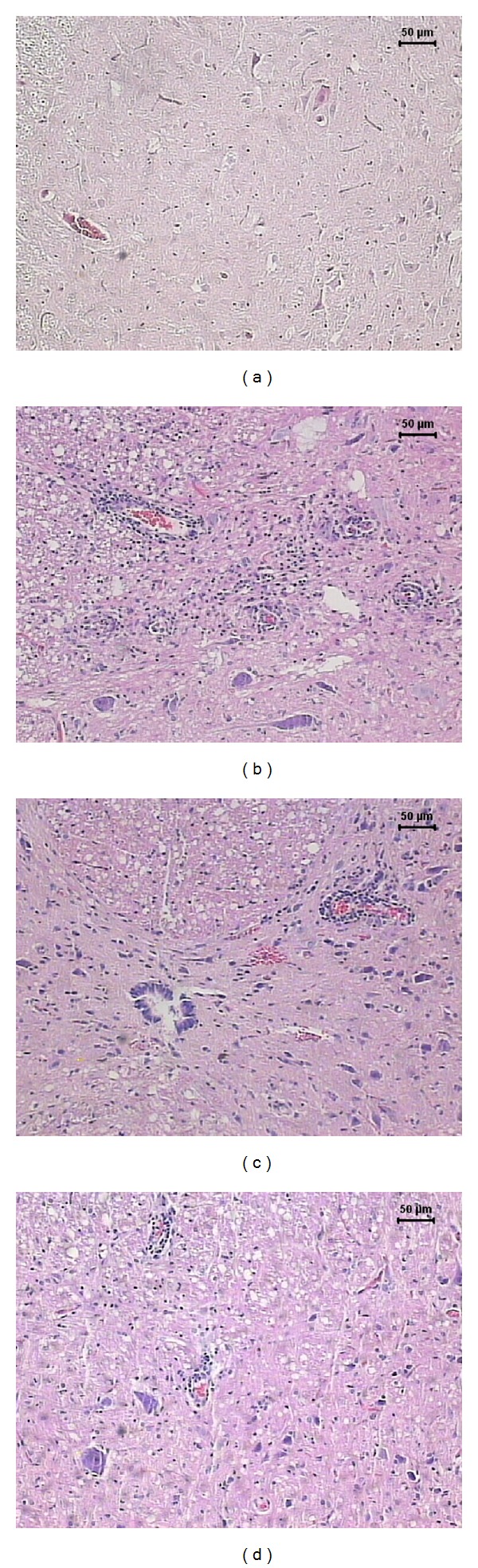
Effect of tuberculosis vaccines in spinal cord inflammation associated with EAE. Female Lewis rats were immunized with BCG alone or with BCG followed by pVAXhsp65 boosters and then underwent EAE induction by inoculation of MBP emulsified with CFA. Animals were euthanized during the recovery phase (20th day after MBP injection) and the lumbar spinal cord was removed and further stained by haematoxylin and eosin. Normal control (a), EAE control (b), rats immunized with BCG before EAE (c), and rats immunized with BCG/pVAXhsp65 before EAE induction (d).

The severity of the inflammatory reaction in the CNS from the EAE group (positive control) clearly correlated with the observed clinical symptoms. Sections from the brain and lumbar spinal cord showed typical inflammatory foci, dominated by mononuclear cells that were localized around small vessels, as can be observed in Figures 2(b) and 3(b), respectively. As expected, control animals without EAE did not present any inflammatory foci in the brain (Figure 2(a)) and lumbar spinal cord (Figure 3(a)). However, a previous vaccination with BCG alone (Figure 2(c)) or combined with pVAXhsp65 (Figure 2(d)) or pVAX, the empty vector (not shown), clearly decreased inflammation at the brain in comparison to the EAE group (positive control). These results were further ascertained by a morphometric analysis, which indicated the presence of significantly lower inflammatory infiltrates in animals immunized with BCG alone or combined with pVAXhsp65, in comparison to the EAE group (positive control) (Table 1). The previous immunization with BCG, associated or not with pVAXhsp65, did not affect the intensity of inflammation in the lumbar spinal cord as demonstrated in Figures 3(c) and 3(d) and in Table 1.

### 3.3. Production of IFN-*γ* and IL-10

**Figure 4:**
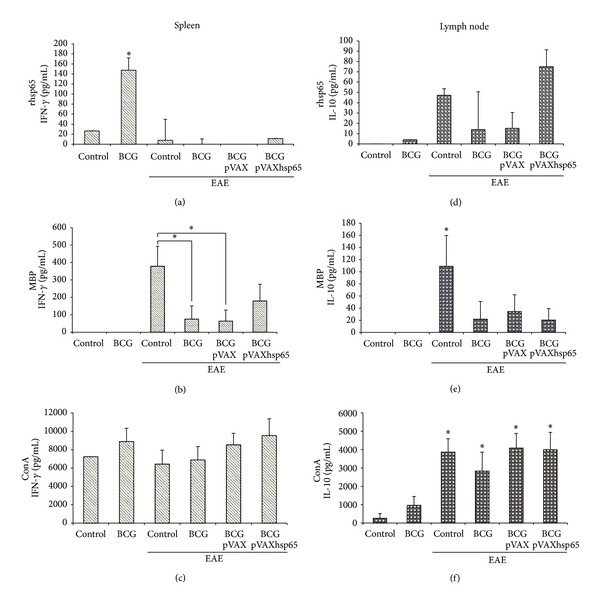
Effect of tuberculosis vaccines in IFN-*γ* and IL-10 production. Female Lewis rats were immunized with BCG alone or with BCG followed by pVAXhsp65 boosters and then underwent EAE induction by inoculation of MBP emulsified with CFA. Animals were euthanized during the recovery phase (20th day after MBP inoculation) and spleen and lymph node cell cultures were stimulated with rhsp65 ((a) and (d)); MBP ((b) and (e)); and ConA ((c) and (f)). IFN-*γ* levels were evaluated in spleen cell cultures ((a), (b), and (c)) and IL-10 levels were evaluated in lymph node cell cultures ((d), (e), and (f)). Data are presented by mean ± SEM for 4–6 animals. *Represents the difference between immunized and control groups. *P* < 0.05.

The highest IFN-*γ* levels, in cultures stimulated with rhsp65, were found in the BCG immunized group. In all experimental groups with EAE, independently of a preceding immunization, IFN-*γ* levels were very low, that is, similar to the normal control group (Figure 4(a)). As can be observed in Figure 4(d), IL-10 levels in rhsp65 activated cultures presented a very distinct pattern: the highest levels were found in EAE and BCG/pVAXhsp65 EAE groups. BCG group presented detectable but very low IL-10 levels.

The production of these two cytokines, in cultures stimulated with MBP, followed the same pattern (Figures 4(b) and 4(e)). In this case, the highest levels of both cytokines were found in the EAE group. Previously immunized groups, that were later submitted to encephalomyelitis induction presented lower IFN-*γ* (Figure 4(b)) and IL-10 (Figure 4(e)) levels in comparison to the control EAE group. IFN-*γ* and IL-10 were not detected in the BCG group.

 IFN-*γ* levels were similarly elevated in all groups stimulated with ConA (Figure 4(c)). On the other hand, even though there was some IL-10 production by cultures from the BCG group, the levels of this cytokine were significantly high in all groups with encephalomyelitis, independently of a previous immunization (Figure 4(f)).

## 4. Discussion

In this study, we investigated the effect of a previous immunization with BCG, associated or not with pVAXhsp65, on the development of EAE. This kind of investigation is relevant because a possible connection between vaccines and autoimmune phenomena is surrounded by controversy. Some literature reports indicate that BCG could be one of the vaccines associated with autoimmune diseases. For example, oligo and polyarticular arthritis were detected in approximately 3% of patients with bladder carcinoma that were treated with intravesicular BCG [35]. It is also being described that mycobacteria precipitates an SLE-like syndrome in NOD mice [36].

Otherwise, the maintenance of BCG in a new prophylaxis against TB is highly expected because this vaccine is widely accepted in the developing countries, it has protective effect against the most severe forms of TB in children and it is also endowed with immunomodulatory properties [37–39]. In this scenario, safety aspects need to be experimentally validated.

In the last years, our group analyzed a DNA vaccine containing the hsp65 gene from *M. leprae* (pVAXhsp65). The results showed that this vaccine, by itself or associated with BCG, is able to confer protection in different TB experimental models [22]. This genetic construction includes the mycobacterial hsp65 gene that presents a high homology degree with the corresponding hsp60 mammalian gene. A specific immune response against this bacterial protein could cross-react with the corresponding mammalian protein and, therefore, trigger an autoimmune pathology. 

In this context, the most relevant contribution of this work was the demonstration that the previous contact with BCG or BCG followed by pVAXhsp65 boosters did not deleteriously affect the clinical EAE development in Lewis rats. This was initially demonstrated by the very similar clinical evolution of the disease in vaccinated and nonvaccinated animals; they equally lost weight, the average clinical score was the same, and acute and remission phases occurred at comparable time periods. In addition of this absence of a detrimental effect, the previous immunization with BCG or BCG/pVAXhsp65 also determined a clear anti-inflammatory reaction in the brain. This anti-inflammatory activity did not reach the spinal cord; this could explain the absence of a beneficial clinical effect over EAE clinical score. The mechanism of this differential protective effect over the brain and the spinal cord was not investigated. However, considering that interaction of circulating leukocytes with the endothelium of blood-brain and blood-spinal cord barriers is fundamental in inflammatory pathologies of the CNS and that they differ in many aspects, we could hypothesize that differences in adhesion molecules, chemokines, or their receptors could explain this finding [40].

A similar protective ability of BCG towards autoimmunity was already described. Van Eden et al. in 1988 [41], demonstrated that administration of BCG antigens to rats determined resistance to subsequent induction of adjuvant arthritis. A preceding contact with BCG also determined protection against diabetes in NOD and streptozotocin diabetes models [42, 43]. More recently, in this same line of research, Sewell et al. in 2003 [44], demonstrated that previous immunization with live BCG clearly reduced clinical severity in murine EAE.

The protective ability of hsp65 is even more widely accepted and investigated. Heat shock proteins, especially hsp65, are understood as targets for regulatory T cells due to their enhanced expression in inflamed tissues. There are also very strong evidence that they are able to induce anti-inflammatory T cell responses [45, 46]. Our previous experience indicates that DNAhsp65 had a similar protective effect over arthritis, diabetes, and encephalomyelitis. However, this protective effect was clearly more accentuated in arthritis and diabetes. In these two conditions, its immunomodulatory effect was strong enough to determine clinical improvement [47, 48]. In the case of EAE, DNAhsp65 determined less inflammation but no improvement was detected in clinical scores [49].

Considering this immunoregulatory ability of hsp65, we could expect a more pronounced anti-inflammatory effect in the prime-boost, in comparison to the BCG immunized group. However, these two groups presented similar clinical evolution. This finding is in contrast to our recent experience with a similar prime-boost in NOD mice. In this model, priming with BCG followed by two boosters with pVAXhsp65 prevented pancreas inflammation and clinical diabetes development [50].

The analysis of IFN-*γ* and IL-10 levels, produced by peripheral lymphoid organs, answered some mechanistic questions. We initially asked how BCG decreased inflammation in the brain. In this sense, the most interesting finding was the accentuated drop of IFN-*γ* and IL-10 levels induced by MBP, in EAE experimental groups previously vaccinated, in comparison to the EAE control group. A possible explanation for this finding could be the trapping of nervous tissue-specific T cells in peripheral BCG inflammatory sites as elegantly demonstrated by Sewell et al. in 2003 [44]. Alternatively, these autoreactive T cells could be in lower numbers due to an apoptotic process occurring in the periphery. This phenomenon was clearly demonstrated by O'Connor et al. in 2005 [51]. These authors detected high levels of apoptosis among activated CD4+ T cells in BCG experimental infection. Interestingly and concerning to the model used by us, these authors also described that the high apoptotic degree occurred simultaneously with a milder experimental encephalomyelitis course. Even though we did not observed improvement in clinical parameters, a significant drop in brain inflammation was detected. This lower IFN-*γ* and IL-10 production could also be linked to the migration of myelin-specific T clones to the brain, where they could exert the detected anti-inflammatory activity. IFN-*γ* is described as able to shape the immune infiltration of the CNS by controlling chemokine expression. In addition, this cytokine accentuates apoptosis of infiltrating encephalitogenic T cell clones [52, 53]. Additionally, the production of IL-10 by Tr1 regulatory cells has been widely accepted as one of the mechanisms responsible for MS and EAE downregulation [54].

## 5. Conclusion

These results indicate that immunization procedures with BCG or BCG/pVAXhsp65, as used in this investigation, did not deleteriously affect EAE development.

## Figures and Tables

**Table 1:** Morphometric analysis of perivascular inflammatory infiltrate in the brain and lumbar spinal cord samples from rats immunized with BCG alone or associated with pVAXhsp65 before EAE induction.

	Brain *n* = 4	Lumbar spinal cord *n* = 4
	(*μ*m^2^ of mononuclear infiltrate/mm^2 ^of organ section)^†^	
Control EAE	3.55 ± 0.72	6.56 ± 2.72
BCG EAE	1.17 ± 0.38*	7.38 ± 2.53
BCG pVAXhsp65 EAE	1.50 ± 0.71*	4.46 ± 0.79

*P < 0.05 versus control EAE.

^†^Morphometric analysis was done in 5-micron thick sections after haematoxylin and eosin stain using a Nikon Microphot-FXA optical microscope connected to a computer and employing the KS300 software.
